# EU pharmaceutical expenditure forecast

**DOI:** 10.3402/jmahp.v2.23738

**Published:** 2014-10-30

**Authors:** Duccio Urbinati, Cécile Rémuzat, Åsa Kornfeld, Anne-Lise Vataire, Laurent Cetinsoy, Samuel Aballéa, Olfa Mzoughi, Mondher Toumi

**Affiliations:** 1 Creativ-Ceutical, Milano, Italy; 2 Creativ-Ceutical, Paris, France; 3 Faculté de Médecine, Laboratoire de Santé Publique, Aix-Marseille Université, Université de la Méditerranée, Marseille Cedex, France

**Keywords:** forecast model, pharmaceutical expenditure, health policy, generic, biosimilar, innovative medicine

## Abstract

**Background and Objectives:**

With constant incentives for healthcare payers to contain their pharmaceutical budgets, forecasting has become critically important. Some countries have, for instance, developed pharmaceutical horizon scanning units. The objective of this project was to build a model to assess the net effect of the entrance of new patented medicinal products versus medicinal products going off-patent, with a defined forecast horizon, on selected European Union (EU) Member States’ pharmaceutical budgets. This model took into account population ageing, as well as current and future country-specific pricing, reimbursement, and market access policies (the project was performed for the European Commission; see http://ec.europa.eu/health/healthcare/key_documents/index_en.htm).

**Method:**

In order to have a representative heterogeneity of EU Member States, the following countries were selected for the analysis: France, Germany, Greece, Hungary, Poland, Portugal, and the United Kingdom. A forecasting period of 5 years (2012–2016) was chosen to assess the net pharmaceutical budget impact. A model for generics and biosimilars was developed for each country. The model estimated a separate and combined effect of the direct and indirect impacts of the patent cliff. A second model, estimating the sales development and the risk of development failure, was developed for new drugs. New drugs were reviewed individually to assess their clinical potential and translate it into commercial potential. The forecast was carried out according to three perspectives (healthcare public payer, society, and manufacturer), and several types of distribution chains (retail, hospital, and combined retail and hospital). Probabilistic and deterministic sensitivity analyses were carried out.

**Results:**

According to the model, all countries experienced drug budget reductions except Poland (+€41 million). Savings were expected to be the highest in the United Kingdom (−€9,367 million), France (−€5,589 million), and, far behind them, Germany (−€831 million), Greece (−€808 million), Portugal (−€243 million), and Hungary (−€84 million). The main source of savings came from the cardiovascular, central nervous system, and respiratory areas and from biosimilar entries. Oncology, immunology, and inflammation, in contrast, lead to additional expenditure. The model was particularly sensitive to the time to market of branded products, generic prices, generic penetration, and the distribution of biosimilars.

**Conclusions:**

The results of this forecast suggested a decrease in pharmaceutical expenditure in the studied period. The model was sensitive to pharmaceutical policy decisions.

The evolution of pharmaceutical expenditure is mainly driven by the entrance of new branded products and products going off-patent. The European patent cliff peak of 2011–2012 impacted a large number of blockbuster drugs which had historically been the key growth drivers for the pharmaceutical industry. Because of this, very high sales potential for generic products and important generic competition within Europe are expected in the next decade.

According to IMS Health forecasts (1), spending on medicines in the five major European Union (EU) markets (France, Germany, Italy, Spain, and the United Kingdom (UK)) is expected to grow between −1 and 2% through 2016. The therapeutic classes that are expected to record the highest levels of pharmaceutical spending on medicines in 2016 include oncology, diabetes, and asthma/chronic obstructive pulmonary disease. According to Datamonitor forecasts (2), a slowdown should be expected by 2016, with forecast sales growth at a compound annual growth rate of 1.9% in the five major EU markets. The therapeutic classes expected to be the principal growth drivers by 2016 are oncology, endocrinology, infectious diseases, and immunology/inflammation. Those expected to decrease, even if they remain among the dominant therapy areas, are mainly cardiovascular and central nervous system, due to the patent cliff and the saturation of these markets with me-too drugs.

A number of other factors – such as ageing populations, the growing prevalence of chronic disease, and the greater use of expensive treatments – have impacted, and will continue to impact, pharmaceutical expenditure. According to Eurostat's population projections (3), covering the period from 2011 to 2060, ageing is likely to affect all EU Member States. The increasingly ageing population and changes in lifestyle habits are leading towards a growing prevalence of chronic diseases, expanding the use of new medical technologies and biologic drugs and driving the growth of pharmaceutical expenditure in European markets.

In addition, rising healthcare costs as well as the global economic downturn led healthcare payers to opt for austerity measures such as reductions in healthcare budgets, price and reimbursement cuts, increased use of price negotiations and pharmacoeconomics, together with enhanced generic uptake. Recent regulatory changes emphasize the willingness of governments to reinforce the assessment of drugs’ benefit–risk ratio throughout the products’ life cycle (e.g., the establishment of Risk Management Plans (RMPs) in Europe and the European Directive 2010/84/EU of 15 December 2010 (4) which provides a legal basis to the European Commission and competent authorities to request post-authorization studies on efficacy at the time of granting a drug's marketing authorization, at a later stage, or conditioning the marketing authorization).

It is crucial for EU countries and European regulatory bodies to understand the key drivers of pharmaceutical expenditure and to anticipate the expected baseline evolution of Member States’ pharmaceutical budgets. In light of this, forecasting has become critically important: some countries have even developed pharmaceutical horizon scanning units such as the Health Policy Advisory Committee on Technology (HealthPACT) in Australia (5) or the NIHR Horizon Scanning Centre in the United Kingdom (6).

The objective of this project was to build a model to assess the net effect of the entrance of new patented medicinal products versus medicinal products going off-patent in selected EU Member States. The model was structured on a defined timeframe, taking into account ageing populations, current and future country-specific pricing, reimbursement, generic drug policies, and market access policies.

## Method

### Definition of the scope of the study

The primary stage of this project was to clearly define the scope of the study in terms of the countries to be selected, timeframe, and types of products to be considered.

#### Country selection

This study focused on seven EU Member States’ pharmaceutical budgets: France, Germany, Greece, Hungary, Poland, Portugal, and the United Kingdom. These countries were selected in order to obtain a good representation of the heterogeneity between Member States across the EU. Heterogeneity provides an opportunity to assess how these baseline differences affect budget impact predictions. It also permits researchers to extend the forecasting results to Member States not included in this analysis, and those sharing similar baseline features. The selection of countries was based on several parameters impacting the pharmaceutical budgets: market size, date of entry in the EU, Bismarck versus Beveridge system, and expected heterogeneity based on other indicators such as gross domestic product, healthcare expenditure, available health services, and socio-demographic indicators.

#### Study Timeframe

It was decided to opt for a 5-year forecasting period (2012–2016). This timeframe is commonly used and is a good compromise between the uncertainties related to any forecasting exercise and the need to inform decisions for better planning.

#### Type of products

Branded, biosimilar, and generic medicinal products were considered in this project. Only breakthrough vaccines approved during 2012–2016 were included in the scope of the project. Indeed, as delays in coverage for new vaccines are considerably longer than those for other pharmaceutical products, significant sales were not expected from a non-breakthrough vaccine.

### Development of the pharmaceutical expenditure forecast

The pharmaceutical expenditure forecast model was built in three main steps, under the supervision and validation, at each stage, of a board of six experts with strong experience in market access of healthcare products and healthcare policies.

The first step of this project was to gather from databases country-specific variables in order to identify the main inputs that would feed the model.

Pharmaceutical pricing and reimbursement policies related to brand, generic, and biosimilar products were gathered for each country from publicly available sources,^1^ from local experts and companies (for Greece, Hungary, Poland, and Portugal), and from internal proprietary databases.

In the second step of the project, several databases^2^ were cross-checked to identify the range of products that would go off-patent and those that would enter the market between 2012 and 2016. Products that went off-patent and those that entered the market in 2010 and 2011 were also considered, taking into account their potential impact on the budget of the forecasted period.

New drugs were selected according to the following criteria:

1. Drugs that could be approved or were approved (in 2010 and 2011) via the European Medicines Agency procedure (i.e., drugs for the treatment of HIV, cancer, diabetes, neurodegenerative diseases, immune dysfunctions, and viral diseases; medicines derived from biotechnology processes; advanced-therapy medicines; orphan medicines; or medicines considered to be significant innovations, or whose authorization would be in the interest of public health) (7).
2. Only the first approval of new entities was considered. Generics, renewals, variations, or updates were excluded.
3. Only products that had a positive phase IIb on the primary endpoint were taken into account, in order to minimize uncertainty and be consistent with the time window considered for this project. Orphan drugs with an ongoing phase II, due to the orphan procedures, could potentially reach the market during the forecasted period and were considered as being possible new drugs.

**Fig. 1.**
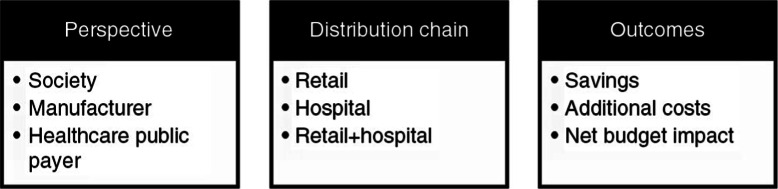
Description of model characteristics.

Finally, the budget impact calculation of generic entries and of new approved innovative pharmaceutical products was based on two separate models and displayed according to three perspectives (healthcare public payer, society, and manufacturer), several types of distribution chains (retail, hospital, and combined retail and hospital), and several outcomes (savings due to products going off-patent, additional costs due to new drugs, and net budget impact) (Fig. 1). The healthcare public payer perspective was the one selected for this article. Drugs’ sales values for 2011 were extracted from the IMS database (note that no reliable sources for hospital sales were available for Greece; however, most drugs are acquired through retail chains).

1. A model for generics and biosimilars was developed for each country. This model estimated the separate and combined effects of direct and indirect impacts on savings from the genericization of the market for each year in the forecasted period. For the retail chain, the evolution of sales of generic and biosimilar products was assumed to be linear until they reached peak sales, considering that the largest budget impact happened after peak sales. Peak sales were assumed to be reached at day 1 for a hospital chain, as hospitals optimize their purchaser positions through tenders. Moreover, hospitals were assumed to use exclusively generic and biosimilar products when available.
2. A model, taking into account the risk of development failure per therapeutic area, was developed for new drugs. It estimated the value of sales and the progression of market share in a competitive environment. The new drugs to insert in the model were reviewed individually by the study board of experts. Their clinical potential was assessed in order to translate it into commercial potential. Peak sales for new drugs were considered to be reached over a period of 3 years, based on the innovative status of the drugs.

Probabilistic and deterministic sensitivity analyses were carried out regarding intrinsic uncertainty surrounding the estimations. Ageing was taken into account in the model (average evolution from 2012 to 2016 was derived from the 2012 ageing report published by the European Commission) (8).

A detailed description of the model is presented in a separate article, ‘Novel methodology for pharmaceutical expenditure forecast’ (9).

The study board of experts assessed the indirect impact of generic entries (e.g., several generics available in the same therapeutic class, or potential changes in recommendation following the entry of a new generic and its consequences) and the impact of new drugs. They attempted to anticipate future regulations for pricing and reimbursement in light of the current trends and ongoing pricing and reimbursement reforms in many EU Member States. The board also validated the most appropriate information to be retained in case of inconsistencies between multiple sources of information.

## Results

### Collection of information regarding specific pharmaceutical policies in place in each country

Extensive changes are seen in the pharmaceutical market access environment of the EU Member States under study. Pricing and reimbursement regulations have shown substantial strengthening trends. The new health bill AMNOG (Arzneimittelmarkt-Neuordnungsgesetz), which was approved at the end of 2010 in Germany, represented a significant change for the introduction of drugs, initiating the concept of benefit–cost assessments of drugs. In France, the new drug safety law approved in 2011 reinforced the economic assessment of health products as well as the provision of real-world data to support the benefit–risk ratio of drugs. Pharmaceutical expenditure containment measures were introduced in 2011 in Greece. These included reducing pricing, increasing generics use, reducing hospital cost, and merging the health insurance function to create a single body, the National Health Services Organization (EOPYY). In Poland, the new reform on pricing and reimbursement of January 2012 introduced health technology assessment and price negotiation. In the United Kingdom, value-based pricing was expected to be introduced in 2014, replacing free pricing, price cuts, price freezes, and profit controls. It will include both pre- and post-launch price reviews, to reflect any changes in a drug's value as a result of new evidence.

A wide variability between countries was found in relation to generics entry policies. Market entry varied from 0 days for the United Kingdom and Germany to 270 days for Greece. Penetration rate was high in all countries (100% for Hungary, and 85–80% for France, the United Kingdom, Germany, and Poland) except for Greece and Portugal (25%).

Generics prices were generally set at a lower level than branded product prices and ranged from the same price as the latter to 60% lower than the branded product at the time of entry. Price reduction versus branded product varied from one country to another (from 45% for Poland to 75% for the United Kingdom). Generic entry led to discounts on brands from 0 to 50%. Price reduction of generics versus branded products (generic price linkage) either is mandatory as per regulatory rules (France, Greece, Hungary, Poland, and Portugal) or depends on the competition level (Germany or the United Kingdom), and it increases over time with generic competition. It is interesting to observe that in Hungary, originator brands are excluded from the market 1 to 1.5 years after generics’ entry, and only generics compete against each other.

It should be brought to notice that pharmaceutical market access policies differ between the inpatient and ambulatory sectors. While there is a tender market for hospitals, with a strong competition among products, the distribution chain and prices are much more regulated for the outpatient market. The hospital market is affected by a large discount practice and could be considered as a de facto 100% generic market. Because of this, generic and biosimilar policies will impact the retail market substantially more.

Little information was available on national pricing and reimbursement policies for biosimilars, despite the fact that Europe is the market with the greatest number of pipeline and marketed biosimilar products, totaling 67 (i.e., the total number of biosimilar product candidates for development, in a development stage (preclinical, clinical trial, or pre-approval), or approved and marketed) and encompassing 32 molecules as of December 2011 (10). Nevertheless, some data about biosimilar penetration were found for France and Germany. These two countries account for half of the biosimilars market by value, with 34 and 17% shares, respectively, across Europe (11). A few policies about prices were reported for Hungary.

Model parameters used to assess the pharmaceutical forecast were based on the data collected and on inputs from the board of experts. Regarding the time to market for new drugs, new drug sales were assumed to impact the markets of all countries (except for Germany) the year after the EU date of marketing approval, due to pricing and reimbursement policies. For Germany, the impact was assumed to be the year of EU launch, as companies initiate sales based on free pricing. Manufacturers have to apply a negotiated price, in the first year following market approval, if an added value is found compared to the reference comparator designated by the Federal Joint Committee (G-BA) (Table I).

**Table:** *Table 1*. Model parameters used to assess the pharmaceutical forecast

	France	Germany	Greece	Hungary	Poland	Portugal	United Kingdom
Brands							
Time to market after marketing authorization (months)	12	0	12	12	12	12	12
Generics: retail chain							
Time to market after marketing authorization (days)	60	0	270	45	180	150	0
Price reduction of the generic versus the original branded product (%)	60	55	60	55	45	60	75
Generic penetration (generics and off-patent brands): volume uptake (%)	80	85	25	100	85	25	80
Time to reach maximum of generic penetration (months)	36	12	36	18	24	30	12
Impact of generic entry on brand price (%)	20	0	50	0	25	0	0
Generics and biosimilars: hospital chain							
Time to market after marketing authorization (days)	0	0	0	0	0	0	0
Price reduction of the generic or biosimilar versus the original branded product (%)	80	80	80	80	80	80	80
Generic and biosimilar penetration (generic, biosimilar, and off-patent brands): volume uptake (%)	100	100	100	100	100	100	100
Time to reach maximum of generic or biosimilar penetration (months)	0	0	0	0	0	0	0
Impact of generic or biosimilar entry on brand price (%)	0	0	0	0	0	0	0
Biosimilars: retail chain							
Time to market after marketing authorization (days)	470	180	450	580	540	530	180
Price reduction of the biosimilar versus the original branded product (%)	30	25	25	50	45	30	25
Biosimilar penetration (biosimilar and off-patent brands): volume uptake (%)	15	25	5	100	25	15	15
Time to reach maximum of biosimilar penetration (months)	36	12	36	18	24	30	12
Impact of biosimilar entry on brand price (%)	10	0	25	0	12	0	0
Reimbursement							
Reimbursement rates (%)	69	90	80	67	62.5	81.6	100

### Identification of products of interests

After investigation, no vaccine was considered to have a significant budget impact in the 5-year period of interest.

Seventy-one products that went off-patent and 66 new drugs were identified during the period of 2010 and 2011, and 202 generics, 10 major biosimilars, and 254 new drugs during the period of 2012–2016.

### Budget impact analysis

**Fig. 2.**
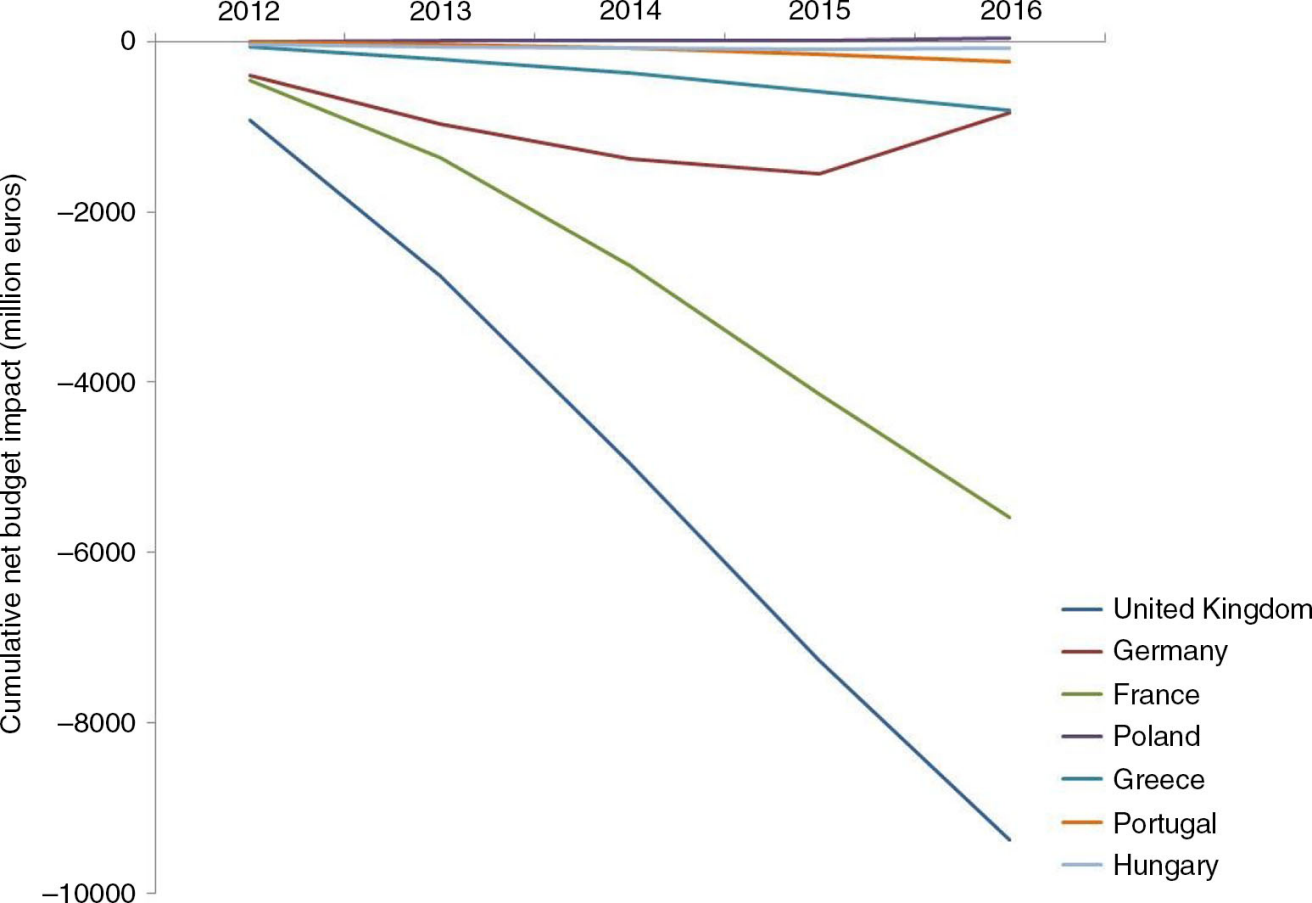
Cumulative net budget impact per year (millions €) and per country from the healthcare public payer perspective.

The budget impact analysis showed that during the period of interest, all countries would experience a drug budget reduction, except Poland which would experience an increase of +€41 million. The decrease in drug expenditure was the highest for the United Kingdom with −€9,367 million, followed by France with −€5,589 million, and followed far behind by Germany with −€831 million, Greece with −€808 million, Portugal with −€243 million, and finally Hungary with −€84 million (Fig. 2 and Table II).

**Table:** *Table II*. Net pharmaceutical budget impact during 2012–2016 per country from the healthcare public payer perspective (millions €)

Country	Net pharmaceutical budget impact during 2012–2016 (millions €)
France	−5,589
Germany	−831
Greece	−808
Hungary	−84
Poland	+41
Portugal	−243
United Kingdom	−9,367

**Fig. 3.**
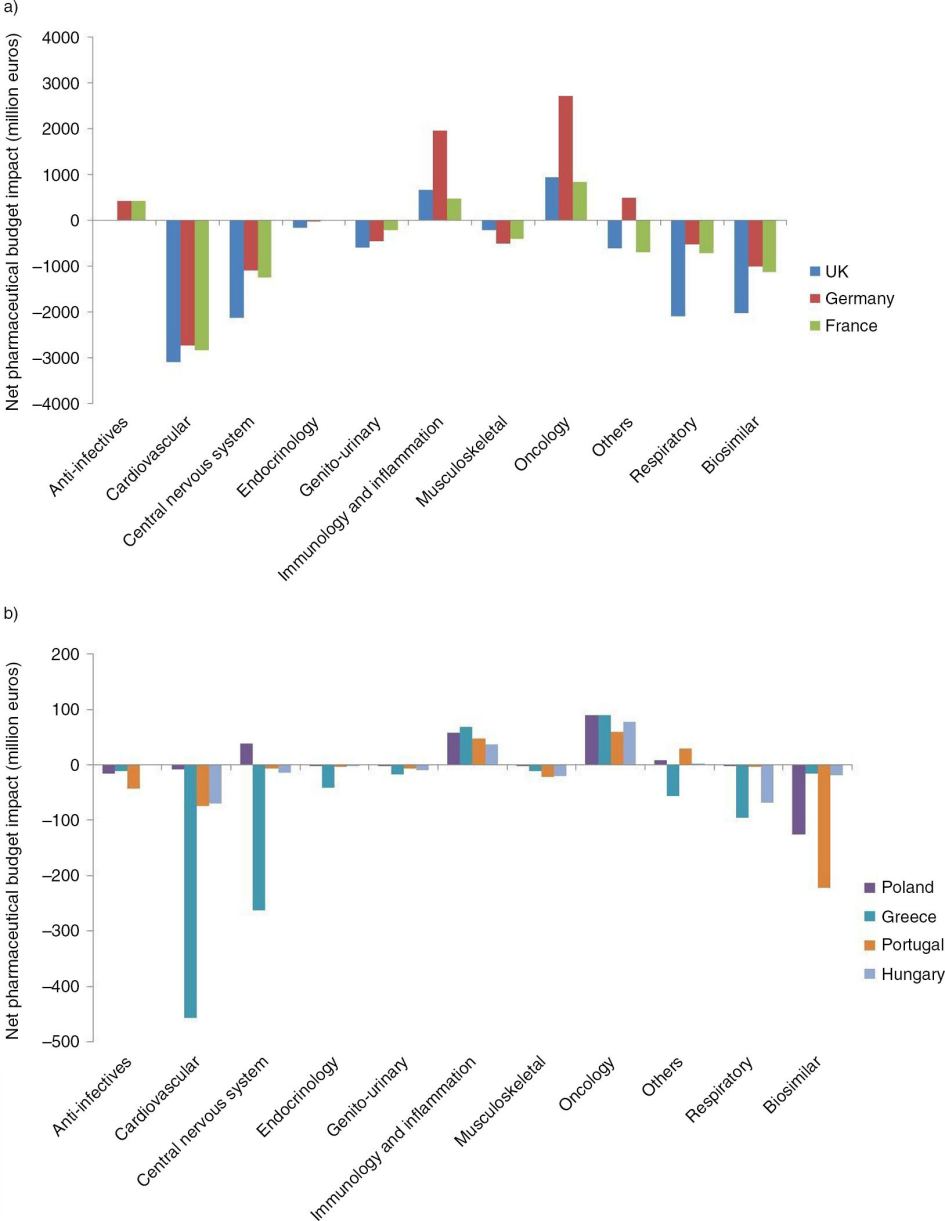
Net pharmaceutical budget impact (millions €) during 2012–2016 per therapeutic class from the healthcare public payer perspective for (a) the United Kingdom, Germany, and France; and (b) Poland, Greece, Portugal, and Hungary.

The budget analysis per therapeutic area indicated that the cardiovascular and central nervous system areas accounted for the most important savings. Values varied from one country to another, with budget impacts ranging from −€3102 million in the United Kingdom to −€8 million in Poland for the cardiovascular area. For the central nervous system area, the budget impact ranged from −€2,137 million in the United Kingdom to +€38 million in Poland. Those areas were followed by the respiratory area (−€2,093 million/UK to −€2 million/Poland). Biosimilar entry also accounted for important savings from −€2,023 million for the United Kingdom to −€15 million for Greece. As for additional costs, oncology was the leading source, with expenditure from +€60 million in Portugal to +€2,714 million in Germany. Oncology was followed far behind by immunology and inflammation (expenditure from €37 million/Hungary to €1,952 million/Germany) (Fig. 3a and 3b).

Deterministic one-way sensitivity analysis showed the great influence of the price reduction of small-molecule generics versus branded products and of the penetration rate of these generics via the retail chain distribution. It also highlighted the importance of the time to market of new branded products as a critical factor for budget impact. In addition, this analysis indicated that the impact of biosimilar savings was critically affected by the proportion of hospital distribution.

## Discussion

The forecast exercise predicted that the expenditure were likely to decrease, except for Poland, during the period 2012–2016 due to three main factors. Firstly, even if the phenomenon of genericization tends to slow down around the end of 2016, it will be progressively replaced by the arrival of biosimilars. It will be interesting to observe the important savings that biosimilars will probably determine on the global drug budget, in Europe, beyond 2016. Secondly, the criteria to assess the added value of the new drug products were more and more severe in recent years, and this trend will likely escalate in the future. Indeed, this point raises the issue of innovation's reward and incentive for developing new products. A third reason is the reduction of approvals of new entities that bring additional benefits to existing alternatives. This trend is likely to change with the development of biologics that are expected to reach the market in the medium to long term.

The Polish and Hungarian markets are still far from being mature markets and will certainly, in the future, increase their investment to secure patient access to innovative products. In the five other countries, the market is quite mature and has a thinner margin of progression. Moreover, Portugal and Greece are today widely impacted by the economic crisis, which will act as a brake to potential progression of the market.

During the study period, unsurprisingly, the therapeutic areas that drove up health expenditure were oncology, immunology, and inflammation. These disease areas are the ones with high unmet needs for which new biologic entities are expected to enter the market with substantial clinical benefits. Other important areas considerably impacting the budget were the cardiovascular, central nervous system, and respiratory areas, with an overall negative net budget impact, as more savings will occur in relation to generic entry than additional costs related to new brands.

It is generally hard to establish the exact patent expiration date for biosimilar drugs. Because of this, significant biologicals’ expiration dates during the forecasted period were mainly extracted from the most recent publicly available data from the Generics and Biosimilars Initiative (GaBi) (12). Only European expiry dates could be identified for these molecules (except for one product with a specific launch date found for the United Kingdom). On another point, as the approval of some molecules under the ‘generics regulations’ or the ‘biosimilars regulations’ was still not obvious (13–15), low-molecular-weight heparin (LMWH) and glatiramer were included among the model's ‘generics’. Also, some biosimilar drugs might not have been taken into account in the considered forecasted period.

Another limitation of this study was that the market development of products launched before 2010 was not considered. These products were not expected to become generic in the considered period. These products were likely to have some potential impact by increasing or decreasing their market shares. However, this was expected to have a low impact on the study results due to the fact that it could lead to an increase as well as decrease in the pharmaceutical budget.

As a last point, the seven European countries selected in the scope of this project were very heterogeneous in terms of level of pharmaceutical expenditure, generic market penetration rates, and public policy approaches towards the price regulation of branded and off-patent pharmaceutical products. They were also different from healthcare service organizations’ and healthcare funding policies’ points of view. This allowed us to capture a broader perspective of the net budget impact of different country's profiles and associated drivers. However, despite all of this, the results might not necessarily be representative of all EU countries.

Finally, the modeling exercise was based on several assumptions that were described in the article ‘Novel methodology for pharmaceutical expenditure forecast’ (9).

## Conclusion

The results of this forecast project suggested a consistent, but variable in magnitude, reduction in pharmaceutical expenditure in all countries with the exception of Poland for the 2012–2016 period. This model was very sensitive to the time to market for branded products, generic prices, generic penetration, and the distribution of biosimilars.

## Footnotes

1 National government websites (publication of applicable laws), national drug agencies, national health technology assessment organization websites, the European Federation of Pharmaceutical Industries and Association (EFPIA) website, the European Generic Association (EGA) website, country-specific pharmaceutical industries associations’ websites, country-specific generic associations’ websites (as well as direct contacts with some of them), the WHO Collaborating Centre for Pharmaceutical Pricing and Reimbursement Policies, the European Commission website, the Pharmaceutical Health Information System database, and the International Society for Pharmacoeconomics and Outcome Research Global Health Care Systems Road Map.

## Footnotes

2 Datamonitor reports; the PharmaVitae database; the Medtrack database; free-access patent databases such as the World Intellectual Property Organization, the European Patent Office, and Espacenet; commercial online databases such as STN International/CAPADOC, Questel Orbit, and Genericsweb; pharmaceutical company websites; press releases; investors’ reports; the IMS database; the Generics and Biosimilars Initiative (GaBi) website; European Medicines Agency public information; study registries such as clinicaltrial.gov; and an in-house proprietary drug information database.
